# Breast Cancer Management Timelines in a Tertiary Care Center During the COVID-19 Pandemic, Makkah City, Saudi Arabia: A Retrospective Study

**DOI:** 10.7759/cureus.42893

**Published:** 2023-08-03

**Authors:** Abdulrahman H Alhassani, Abdulmohsen S Alqurashi, Turki H Alhassani, Sarah M Fageeh, Mohammad I Almatrafi, Emad K Alsharif, Abdulaziz M Alzahrani, Roaa A Attieh

**Affiliations:** 1 General Surgery, Makkah Healthcare Cluster, Makkah, SAU; 2 Faculty of Medicine, Umm Al-Qura University, Makkah, SAU; 3 Division of Breast and Endocrine Surgery, Specialized Surgical Unit, King Abdullah Medical City, Makkah, SAU

**Keywords:** skipped appointments, patient deposition, timelines of management, covid-19, breast neoplasms

## Abstract

Background: Breast cancer (BC) is a prevalent form of cancer and a leading cause of death among women worldwide. In Saudi Arabia, it accounted for 31.8% among females of all new cancer cases reported in 2018. Following the declaration of COVID-19 as a global pandemic, there was a complete redistribution of healthcare resources to face this crisis, which caused a significant delay in the management of various diseases, including BC. There is currently a lack of research in our region on the facility time interval in BC management. Therefore, this study aimed to fill this gap by determining the timelines of diagnosis, management, and factors influencing the delay.

Methods: This observational retrospective study included all female patients diagnosed with BC at or referred to King Abdullah Medical City (KAMC) in Makkah, Saudi Arabia, between January 2020 and August 2021. The data for this study were obtained from a centralized electronic chart review of all included patients at the KAMC center.

Results: A total of 76 patients were included in the study, with a mean age of 50 ± 11 years. In terms of the disease management duration, 20 patients (26.3%) completed their management within 30 days, 28 patients (36.8%) had a management duration between 31 and 60 days, and the management duration of 28 patients (36.8%) exceeded 60 days. Patient deposition showed a significant association with delay (p = 0.033). A higher incidence of delays at the initiation of treatment was observed in patients who failed to attend appointments (p < 0.001). Among patients who skipped two or more appointments, 12 individuals (80%) experienced a delay of more than 60 days. Moreover, appointment cancellation was associated with delayed treatment initiation (p = 0.03). Patients' age and comorbidity showed no significant association (p = 0.49, p = 0.24, respectively).

Conclusion: Our findings highlight the significant impact of patient deposition and canceled or skipped appointments on delayed initiation of therapy for BC patients. Further research should be conducted to evaluate the impact of COVID-19 on other malignancies.

## Introduction

Breast cancer (BC) is the predominant form of cancer and a primary contributor to female mortality worldwide. According to the Saudi Cancer Registry, In Saudi Arabia, it accounted for 31.8% among females of all new cancer cases reported in 2018 [1-3]. The asymptomatic nature of this form of cancer is one of the key factors leading to 50% of patients being diagnosed at an advanced stage, subsequently resulting in a higher mortality rate. Early detection enhances the chance of survival and raises the possibility of a cure by over 95%, reducing mortality by up to 30% [4].

The emergence of the new coronavirus disease in 2019 (COVID-19) has led to the implementation of several measures to minimize the transmission of the disease. Here in Saudi Arabia, all educational institutions, social gatherings, sporting events, domestic travel, and international flights were suspended following the disclosure of the first case. Before the first 100 confirmed COVID-19 cases, restrictions on social gatherings, travel, companies, and other activities were put in place [5]. Patients with fever or suspected respiratory symptoms are hospitalized preferentially. Consequently, some healthcare facilities had to postpone non-urgent appointments, reduce the availability of hospital beds and the capacity of the operating rooms, and delay elective surgeries and surgical interventions. Moreover, apprehension among certain patients further contributed to their reluctance to seek hospital care. Nevertheless, these arrangements impeded patients' responses to other diseases, especially cancer [6-10]. Subsequently, disregarding and prolonging the time to seek medical advice from the initial presentation and the underlying reasons for the delay played a significant role in the emergence of advanced and incurable cases with elevated mortality rates [2,11,12].

Time to surgery (TTS) serves as an important metric in evaluating the quality of care in BC treatment. Prolonged delays in surgeries have been associated with reduced therapy response rates, as evidenced in previous research [13]. Therefore, it is imperative to identify these factors, as they play a pivotal role in promoting public awareness regarding early detection when the disease exhibits a high degree of treatment responsiveness [4]. Findings from a previous study indicated a 50% increase in the timeline for initiating the initial BC treatment during the COVID pandemic compared to the pre-COVID era. Furthermore, the duration from the onset of symptoms to the diagnosis was significantly extended, with an average increase from 14 days to 42 days [9]. A study conducted in China during the COVID-19 pandemic revealed a 20% increase in the duration between diagnosis and the initiation of the first treatment [10]. Several geographical and facility-specific variations have been observed, which may be attributed to strategic adaptations to cope with the challenges posed by the pandemic. Nevertheless, there is currently a lack of studies in our region that have measured the facility time interval for BC. Therefore, this study aimed to assess the timelines of diagnosis and management, as well as factors associated with delays. Furthermore, the study investigates the influence of factors specific to the COVID-19 pandemic on the delay in diagnosis.

## Materials and methods

Study design, settings, and participants

This observational retrospective study included female patients diagnosed with BC (confirmed by histopathology) at or referred to King Abdullah Medical City (KAMC) in Makkah, Saudi Arabia, between January 2020 and August 2021. Patients who had received chemotherapy before referral to our center, as well as patients who were scheduled for palliative care, were excluded from this study.

This study was conducted after receiving ethical approval from the Institutional Review Board (IRB) of KAMC (IRB number: 21-871 and registration number: H-02-K-001). All data were safely maintained at the principal investigator's workstation, and all analyses were carried out on-site according to the Declaration of Helsinki, 2013.

Objective of the study

The primary objective of the study was to track the timeline of patients with BC, specifically focusing on three key time intervals, including the duration from the first visit to KAMC, the investigation period, and the time of initiation of individualized treatment plans. Subsequently, we aimed to examine the factors contributing to the delay of patient and/or facility procedures encountered during the COVID-19 era.

Data source and measurements

The study data were obtained through a centralized electronic chart review of all patients included in this study, conducted at the KAMC center. Data on demographic characteristics and comorbidities were collected in accordance with the defined outcome variables. A comprehensive collection of patient records included information on referral type, work-up management (including mammogram, breast ultrasound, breast magnetic resonance imaging (MRI), and biopsy specimen), diagnosis, and histopathology reports, as well as subsequent management plans (including surgery, chemotherapy, hormonal therapy, or radiotherapy). A tracking model for clinic appointments was applied to determine the number of canceled/skipped appointments. Timeline counts for the duration of the investigation and management were calculated in days for each patient. In the context of this research, we established the first visit to our center as the time when patients initially sought evaluation or were referred to our clinic (which included the general surgery clinic, BC clinic, or oncology clinic) to address breast-related complaints.

Statistical analysis

Microsoft Excel (Redmond, USA) was used to gather, manage, and visualize the data. It was then transferred to the statistical software, IBM Corp. Released 2017. IBM SPSS Statistics for Windows, Version 25.0. Armonk, NY: IBM Corp., for data analysis. Frequencies and percentages were used to describe categorical data. For continuous data (including age and duration of investigation), mean and standard deviation (SD) were used for normally distributed data, while median and interquartile range (IQR) were used for skewed data.

The Pearson Chi-square test was applied to evaluate the factors associated with delay in treatment (including age, referral type, comorbidities, skipped appointments, and canceled appointments). A p-value of less than 0.05 was considered statistically significant.

## Results

Seventy-six patients, with an average age of 50 ± 11 years, were included in this study. A total of 47 patients (61.8%) were referred from other centers. Approximately one-third of the patients were hypertensive, and 17 (22.4%) of them had diabetes. Regarding the tumor, node, and metastasis (TNM) stage of the patients, the majority were classified as T2, N1, and M0, accounting for 27 (35.5%), 28 (36.8%), and 74 (97.4%) patients, respectively. The surgical approaches employed in this study included a modified radical mastectomy, partial mastectomy, simple mastectomy, and skin-sparing mastectomy performed on 41 (54%), 23 (30.3%), 11 (14.5%), and one (1.3%) patients, respectively (Table 1).

**Table 1:** Socio-demographic and disease characteristics of study participants SD: Standard deviation; n: Number of patients; CHF: Congestive heart failure; TNM: Tumor node metastasis.

Characteristics	Value
Age in years, mean ± SD	50 ± 11
Referral centers n (%)	
Non-referred patients	29 (38.2)
Referred patients	47 (61.8)
Medical illnesses n (%)	
Diabetes mellitus	17 (22.4)
Hypertension	24 (31.6)
Asthma	4 (5.3)
Hypothyroidism	4 (5.3)
Ischemic heart disease	4 (5.3)
Cirrhosis	2 (2.6)
Chronic kidney disease	1 (1.3)
CHF	1 (1.3)
Adrenal insufficiency	1 (1.3)
TNM stage n (%)	
T	
T0	12 (15.8)
T1	22 (28.9)
T2	27 (35.5)
T3	7 (9.2)
T4	8 (10.5)
N	
N0	27 (35.5)
N1	28 (36.8)
N2	11 (14.5)
N3	10 (13.2)
M	
M0	74 (97.4)
M1	2 (2.6)
Surgical approach n (%)	
Modified radical mastectomy	41 (54)
Partial mastectomy	23 (30.3)
Simple mastectomy	11 (14.5)
Skin- or nipple-sparing mastectomy	1 (1.3)

The duration of the diagnostic workup was measured in days. The study found that non-referred patients or those referred based on suspicion (without biopsy) had a median time of 19 days (IQR: 14 to 29) until the final diagnosis. However, in the referred patients, the median time from the referral to the final diagnosis was 10 days (IQR: 7 to 16). While performing a breast ultrasound and MRI, the median time was 14 days (IQR: 8.25 to 21.5) and 31 days (IQR: 21 to 150) (Table 2).

**Table 2:** Duration of diagnostic establishment IQR: Interquartile range; n: Number of patients; MRI: Magnetic resonance imaging.

Parameter	Median (days) [IQR]
Duration between referral and initial hospital visit (n=47)	7 [4 to 14]
Duration between initial visit and performance of mammogram (n=60)	13 [8 to 18]
Duration between initial visit and performance of breast ultrasound (n=60)	14 [8.25 to 21.5]
Duration between initial visit and performance of breast MRI (n=19)	31 [21 to 150]
Duration between initial visit and performance of biopsy (core or true cut) (n=35)	13 [8 to 24]
Time required to receive biopsy specimen in referred patients who underwent biopsy outside the center(n=41)	4 [2 to 9]
Time required for final diagnosis by biopsy (n=76)	14 [8 to 24]
Time required from first hospital visit to final diagnosis in non-referred patients and patients referred under suspicion (without biopsy), (n=35)	19 [14 to 29]
Time required from first hospital visit to final diagnosis in referred patients with biopsy performed outside the center (n=41)	10 [7 to 16]

In terms of the overall duration of management, 20 patients (26.3%) received treatment within 30 days, while for 28 patients (36.8%), the duration of treatment ranged between 31 and 60 days; additionally, for 28 patients (36.8%), the duration exceeded 60 days. However, when considering the time duration between the initial hospital visit and surgical management, 17 patients (56.7%) underwent breast surgery after more than 60 days. In addition, neoadjuvant chemotherapy was initiated within 30 days for 16 patients (41%) from the initial hospital visit and within 31-60 days for 16 patients (41%) (Table 3).

**Table 3:** Duration of management (days) n: number of patients.

Parameter	0 to 30 days n (%)	31 to 60 days n (%)	More than 60 days n (%)
Duration between initial hospital visits and first management (surgery, chemo, or hormonal, radiotherapy)	20 (26.3)	28 (36.8)	28 (36.8)
Duration between initial hospital visits and surgical management (n=30)	3 (10)	10 (33.3)	17 (56.7)
Duration between initial hospital visits and neo-adjuvant chemotherapy (n=39)	16 (41)	16 (41)	7 (17.9)
Duration between completion of neo adjuvant chemotherapy and surgery (n=37)	13 (35.1)	17 (46)	7 (18.9)
Duration between surgery and adjuvant chemotherapy (n=15)	0 (0)	2 (13.3)	13 (86.7)
Duration between surgery and adjuvant radiotherapy (n=65)	3 (4.6)	34 (52.3)	28 (43.1)

We found no significant association between age and delayed initiation of the first BC treatment (p = 0.49). On the other hand, the patients' deposition (referred versus non-referred) was significantly associated with the delay. Among the non-referred patients, 14 individuals (48.3%) had their treatment initiated 60 days after the diagnosis, whereas among the referred patients, 17 individuals (36.2%) had their treatment initiated within 30 days of the diagnosis (p = 0.033). The patients who skipped appointments experienced a significant delay in the initiation of their treatment (p < 0.001). Among patients who skipped two or more appointments, 12 individuals (80%) experienced a delay of more than 60 days. Additionally, the cancellation of appointments was found to be associated with delayed treatment initiation (p = 0.03) (Table 4).

**Table 4:** Factors associated with delay in initiation of the first breast cancer treatment n: Number of patients.

Parameter	Category	0 to 30 days n (%)	31 to 60 days n (%)	More than 60 days n (%)	p-value
Age (years)	18 to 40	5 (33.3)	7 (46.7)	3 (20.0)	0.49
	41 to 60	13 (27.7)	15 (31.9)	19 (40.4)	
	> 60	2 (14.3)	6 (42.9)	6 (42.9)	
Patient deposition	Non-referred	3 (10.3)	12 (41.4)	14 (48.3)	0.033
	Referred	17 (36.2)	16 (34)	14 (29.8)	
Comorbidity	0	14 (34.1)	13 (31.7)	14 (34.1)	0.242
	1	4 (30.8)	4 (30.8)	5 (38.5)	
	2 or more	2 (9.1)	11 (50.0)	9 (40.9)	
Skipped appointment	None	12 (44.4)	12 (44.4)	3 (11.1)	< 0.001
	1 appointment	8 (23.5)	13 (38.2)	13 (38.2)	
	2 or more appointments	0 (0)	3 (20)	12 (80.0)	
Cancelled appointments	None	19 (36.5)	18 (34.6)	15 (28.8)	0.03
	1 appointment	1 (6.3)	6 (37.5)	9 (56.3)	
	2–3 appointments	0 (0)	4 (50)	4 (50)	

## Discussion

During the COVID-19 pandemic, healthcare resources were extensively utilized to manage the escalating number of COVID cases. However, resource-demanding diseases, including BC, encountered significant challenges. Consequently, many hospitals during the pandemic were compelled to temporarily suspend routine breast screening and follow-up appointments for patients receiving adjuvant therapy to conserve hospital resources and prevent the spread of infection [14-16]. As part of the KAMC policy to face COVID-19 infection, unnecessary appointments were suspended, elective surgeries were delayed, and in admission orders, we used the COVID-19 check-up list and nasal swap to minimize infection spread.

The treatment delay issue among patients with BC has been impacted by changes in hospital policies, priorities, and capacity to accommodate patients. According to Papautsky’s report, approximately half of BC patients experienced delays in their care during the pandemic [17]. The study’s findings illustrated that the delays were observed in patients whose diagnosis was not confirmed through biopsy at the time of presentation. However, less than one-third of patients received their initial treatment within the first 30 days of the hospital visit, indicating a considerable delay in BC management. Additionally, a significant association was found between the delay in BC management and patient deposition, skipped appointments, and canceled appointments.

Furthermore, it is important to note that the delay in management is not solely attributable to hospitals. For instance, the national cancer database has identified delays in low-income patients [18]. Moreover, several patient-related variables can impede the timely management of the disease, including transportation difficulties, low education levels, dependence on other members, language barriers, inability to take time off work, or existing medical conditions [19]. These factors collectively play a significant role in causing delays in BC management.

In our cohort, the median time from the first hospital visit to the final diagnosis was 10 and 19 days for referred patients and non-referred patients, respectively. These results indicate a shorter duration compared to Kapp's report, where the time from symptom onset to diagnosis was more than 25 days [9]. Similarly, Hawrot's study documented a median duration of 23 days from presentation to histological diagnosis [20]. Additionally, Fortin's study demonstrated that the timing of BC diagnosis and treatment nearly doubled during the pandemic compared to pre-pandemic times [21].

In this study, the majority of patients received their initial management, including surgery, chemotherapy, hormonal therapy, or radiation, within an average period of 31 days after their first hospital visit. Specifically, with regard to surgical resection, more than half of the patients underwent surgery within 60 days of their initial presentation. This is in contrast to Mooghal’s study, which reported an average of 69 days from presentation to surgery [22]. Additionally, Gasparri's study revealed an increase in the time from diagnosis to treatment across various surveyed institutions [10].

A significant association was found between the delay in initiating the first management and various factors, including patient deposition and skipping and canceling appointments. This observation sheds light on the potential impact of COVID-19 on both patient- and facility-related management delays. Furthermore, these two factors could help explain the differences in patient presentations before and after the COVID-19 pandemic, as reported in the literature [20,21]. However, it is worth noting that other variables, such as age and comorbidity, did not show a statistically significant association in this study.

To the best of our knowledge, this is the first study to examine BC timeline management in tertiary hospitals during the COVID-19 pandemic in Saudi Arabia. However, there are certain limitations to consider. First, the study design is retrospective, which may restrict the available information and introduce potential biases. Second, the small cohort size could have influenced the results, and a larger sample size would provide more reliable conclusions. Third, the study did not include information on the COVID-19 status of the participants or specific histopathological subtypes. Fourth, we included breast cancer patients who visited KAMC institutions during the COVID-19 era, which is a small group of patients that cannot give a reliable conclusion about factors that contribute to delayed management in KAMC institutions. Further studies should aim to address the impact of COVID-19 on other malignancies and consider a prospective design to gather more comprehensive data.

## Conclusions

In conclusion, this study provides valuable insights into the impact of COVID-19 on the timeline management of BC. Our findings indicate that the delay in initiating therapy for patients with BC was significantly associated with 40 patient depositions, appointment cancellations, and skipped appointments. However, further research is needed to validate these findings on a larger scale, preferably through a nationwide study involving multiple centers, and further studies are needed to address the reasons for delayed management. Additionally, future studies to assess the extent of cancer up-staging caused by these delays should be conducted. These efforts will contribute to a better understanding of the challenges faced by BC management during the COVID-19 pandemic and help guide the development of strategies to mitigate these delays.
